# The Role of Surgery in Oligometastatic Retroperitoneal Sarcoma

**DOI:** 10.3390/curroncol30060398

**Published:** 2023-05-24

**Authors:** Lindsay A. Janes, Christina V. Angeles

**Affiliations:** 1 Department of Surgery, University of Michigan, Ann Arbor, MI 48109, USA; 2 Rogel Cancer Center, University of Michigan, 1500 E. Medical Center Drive, 6219 Cancer Center Ann Arbor, Ann Arbor, MI 48109, USA

**Keywords:** retroperitoneal sarcoma, surgery, metastasis

## Abstract

Retroperitoneal sarcomas are extremely rare, comprising <15% of primary sarcomas. Distant metastasis occurs in about 20% of cases, with pulmonary and hepatic metastasis as the most common sites of hematogenous spread. Although surgical resection is well established as the main treatment of localized primary disease, there are limited guidelines for the surgical treatment of intra-abdominal and distant metastases. There are inadequate systemic treatment options for patients with metastatic sarcoma, thereby necessitating the consideration of surgical options in carefully selected patients. Key points to consider include tumor biology, patient fitness and co-morbidities, overall prognosis, and goals of care. Multidisciplinary sarcoma tumor board discussion for each case is an essential practice in order to deliver the best care to these patients. The purpose of this review is to summarize the published literature on the past and present role of surgery in the treatment of oligometastatic retroperitoneal sarcoma in order to inform the management of this difficult disease.

## 1. Introduction

Soft tissue sarcomas are rare cancers, comprising less than 1% of cancer cases documented annually [1]. Of those, retroperitoneal sarcomas (RPS) are extremely rare, comprising <15% of primary sarcomas [2,3]. Given the low occurrence rates and the large variety of histopathologic presentations (over 70 different subtypes) and different treatment considerations compared to extremity sarcomas, a standard of care for the treatment of retroperitoneal sarcoma is not well established [4]. This is especially prominent in the circumstance of metastatic retroperitoneal sarcomas. The standard treatment recommendation for primary retroperitoneal sarcoma is surgical resection. Even in the setting of large, aggressive histologies of RPS with a high risk of recurrence, surgery is the mainstay given the lack of adequate benefit from systemic therapies. With this modest RPS disease control with current systemic options, surgery is a treatment strategy used for metastatic disease. Although there have been efforts to establish treatment guidelines for metastatic retroperitoneal sarcoma, there are limited primary studies in the literature investigating the role of surgery in treatment outcomes. In this review, we discuss current treatment options and the potential role of surgery in the management of metastatic retroperitoneal sarcomas.

## 2. Clinicopathological Considerations

### 2.1. Characteristics of RPS

The primary location of soft tissue sarcoma is an important prognostic factor, as retroperitoneal sarcomas have a worse prognosis with an increased risk of local recurrence compared to those of the extremity. This is attributed to tumors of the extremity having a smaller size at presentation and the ability to resect or amputate a tumor with a wider compared to the larger size, and to considerations for critical nearby organs in the resection of retroperitoneal sarcomas [5]. Specific areas of concern in the resection of both primary and intra-abdominal metastatic retroperitoneal sarcomas include vessels such as the superior mesenteric artery (SMA), inferior vena cava (IVC), superior mesenteric vein (SMV) and the portal vein, and critical structures such as the mediastinum, spinal cord, porta hepatis, and kidneys [6]. Metastases most often present in the lung, liver, or intra-abdominally. Intra-abdominal metastasis must be distinguished from local recurrence, as it changes management considerations. There are no clear guidelines to distinguish intra-abdominal metastasis from local recurrence; however, these are typically differentiated radiographically by their distance from the site of the primary resection and the presence of multifocal deposits versus solitary lesions [7,8]. While both extremity and retroperitoneal sarcomas metastasize to the lung, retroperitoneal sarcomas have a higher incidence of intra-abdominal and liver metastasis [5].

### 2.2. Histopathology

There is a wide range of histopathologic presentations of retroperitoneal sarcoma. Histopathology is the greatest predictor of disease-specific death, overall survival, local recurrence, and distant metastasis following primary resection in sarcoma patients. This is followed by the completeness of the initial surgical resection, with negative margins leading to better outcomes and higher tumor grades associated with poorer outcomes [9,10,11,12,13,14,15]. The different histologic subtypes have specific patterns of recurrence and metastasis, such as predicting the time and location of spread and response to therapy, which informs the management approach of each patient [15]. In Table 1 we summarize the typical patterns of retroperitoneal sarcoma recurrence and metastasis in order to review the correlation between histology, patterns of metastasis, and overall survival. Based on incidence and prevalence, included are the most common subtypes: well-differentiated liposarcoma (WDLPS), dedifferentiated liposarcoma (DDLPS), leiomyosarcoma (LMS), undifferentiated pleomorphic sarcoma (UPS), malignant peripheral nerve sheath tumors (MPNST), and solitary fibrous tumors (SFT).

### 2.3. Patterns of Recurrence

In the retroperitoneum, liposarcomas and leiomyosarcomas have the highest incidences of primary occurrence [5]. Among the subtypes of liposarcoma, WDLPS is associated with greater rates of disease-free and overall survival. However, both retroperitoneal WDLPS and DDLPS have a high risk of local recurrence following primary resection. WDLPS does not typically metastasize, but it does have the potential to dedifferentiate, and DDLPS has an increased risk of distant metastasis related to tumor grade (10–30%) [9]. Leiomyosarcomas are typically high-grade tumors with an intermediate risk of local recurrence, yet a high risk of distant metastasis [4,10,12,29]. Undifferentiated pleomorphic sarcomas are high-grade tumors with a high risk of both local recurrence and distant metastasis.

In the instance of intra-abdominal or retroperitoneal local recurrence, common practice is to use the time between complete resection and recurrence to ascertain tumor biology. One study showed the median disease-specific survival to be significantly higher (100 months vs. 21 months) in patients with recurrent tumors with a growth rate less than 0.9 cm/month [30]. In patients with recurrent tumors that grew at a more accelerated rate, the disease-specific survival was poor despite aggressive resection. In addition, it is reasonable to monitor patients with intra-abdominal recurrence with a period of observation. During this time, the growth rate of the tumor and identification of additional sites of metastatic disease can be monitored with repeat imaging in 3–6 months and used as an indicator for whether the patient would benefit from another resection. In the setting of high-grade sarcoma recurrence (UPS and grade 2–3 LMS or DDLPS), patients should be considered for systemic chemotherapy while awaiting repeat imaging to determine responsiveness. This same practice has been extrapolated for the surveillance of metastatic disease; however, there is limited evidence supporting or opposing the practice. If the follow-up scans show evidence of widespread or multifocal metastasis, the patient is not considered a candidate for curative surgical resection and has likely been spared from an operation with significant morbidity and which would ultimately not have been curative.

### 2.4. Candidacy for Surgery

Metastatic RPS can be discovered at the time of diagnosis or, more commonly, on surveillance imaging. When patients present with multifocal disease, they are typically considered candidates for palliative surgery for symptom management rather than curative surgery [31]. When a patient with a history of RPS that has previously been resected and now has concerning findings for metastatic disease, consideration should be given to whether a biopsy is indicated. Decision should be based upon the patient’s primary sarcoma histology and whether the lesion’s presentation and imaging findings are consistent with typical patterns of recurrence or metastasis. A biopsy may be necessary in patients with known predispositions to developing secondary cancer (such as Li Fraumeni syndrome or a history of radiation therapy), patients who have known secondary cancers, patients with lesions with inconclusive radiographic features, or cases in which neoadjuvant therapy is being considered [32]. Biopsy and treatment planning should be performed at an institution where there are multidisciplinary sarcoma specialists with tumor board discussions, as this is associated with better outcomes in terms of overall survival of sarcoma patients [8].

Many of the studies on patient factors associated with higher overall survival rates and decreased complications following sarcoma resection were performed on patients who had undergone a primary resection or resection of a local recurrence. In this demographic, patients who present at a younger age and are female have been shown to have higher overall survival rates and decreased morbidity following surgery, as well as those with tumors < 10 cm [33,34]. Patients who present with malnutrition have been shown to have a worse prognosis [35]. Comorbidities impacting a patient’s candidacy to tolerate any surgery, such as cardiopulmonary disease, fitness, and other comorbidities, are also associated with worse outcomes. Frailty index and patient fitness are also considerations in determining whether a patient will have meaningful survival and would benefit from re-resection.

## 3. Location Dependent Considerations in Surgical Management of Metastases

### 3.1. Intra-Abdominal

Almost all published studies on the surgical management of retroperitoneal sarcomas exclude patients with metastatic disease. We will extrapolate from these data to discuss surgical considerations for intra-abdominal oligometastatic disease. A specific concern during RPS resections is renal function, as a nephrectomy may be warranted during resection. Pre-operative renal function should be assessed along with a nuclear renal scan to assess the function of the contralateral kidney. Since there is a significant chance that chemotherapy may be utilized in the metastatic patient, whether nephrectomy will limit therapeutic options should be discussed with the medical oncologist prior to going to the operating room. In patients with intra-abdominal metastasis, the surgeon needs to heavily examine what has been done during the primary and/or previous resections. The surgeon should review prior operative reports if possible, in order to guide operative planning and decision making.

Every subsequent abdominal surgery increases the risk of complications and morbidity. There is increased risk of adhesions among bowel loops, abdominal wall and abdominal organs on each re-operation, which significantly increases the complexity of the surgery. Previous bowel resections and the location of anastomoses are important to know in order to determine the risk of short gut syndrome. There is a risk of short gut when less than 200 cm of small intestine remains; however, if there is >100 cm of small intestine and the colon is in place, the risk of total parenteral nutrition (TPN) dependency is unlikely if nutrition counseling is given [36,37,38]. Additionally, the extent of vascular involvement should be considered. Oncovascular reconstruction is often required for complete RPS resections, with major vessel reconstruction in approximately 15% of RPS resections [14]. While some instances of vascular involvement, such as encasement of the superior mesenteric artery (SMA), deem a tumor unresectable, many named vessels with involvement or encasement could be ligated or reconstructed. These procedures significantly increase the intra- and post-operative risks for the patient and should be reviewed during pre-operative planning. An assessment of the presence of collaterals and the need for possible graft should be considered. The surgeon should have a thorough discussion with the patient regarding the increased risk of thrombosis and need for long term anticoagulation along with the sequela [39]. In the setting of oligometastatic disease, the risks of complex surgery may outweigh the benefits, and these concepts need to be addressed. Intra-abdominal metastectomy is generally not indicated in a patient who presents with multifocal disease, as the operation has significant morbidity and will not likely improve survival.

### 3.2. Pulmonary

For patients who present with pulmonary metastasis (~20% of RP sarcoma patients), metastectomy is generally considered the standard treatment in patients with limited disease outside the lung, a few number of pulmonary metastases, and baseline adequate pulmonary function, with the medical fitness to tolerate the operation [40]. Significant predictors of overall survival are the completeness of resection, histology and grade of the tumor, and age [41]. There are no randomized control trials comparing overall survival between patients who do and do not undergo pulmonary resection for metastatic sarcoma, but pulmonary metastectomy has been correlated with survival benefit [42]. Leiomyosarcoma is the most frequent subtype of lung metastases of RPS, followed by liposarcoma. Neoadjuvant treatments such as radiation therapy and chemotherapy have not been shown to improve overall survival. In the instance of patients who have pulmonary and extrapulmonary metastasis, retrospective data suggest an improved survival benefit in those who undergo pulmonary metastectomy; however, this may be skewed by the careful selection of patients who are candidates for pulmonary metastectomy. Median survival following pulmonary metastectomy is 36.2 months, whereas for those who present with pulmonary metastasis and do not undergo resection median survival is 11 months [41,43]. Again, this large discrepancy is thought to be partially due to the careful patient selection for pulmonary metastectomy.

## 4. Role of Non-Operative Management in the Treatment of Metastatic Retroperitoneal Sarcoma

### 4.1. Radiation

Radiation therapy is utilized in ~25% of primary sarcoma cases, primarily extremity sarcomas, with randomized clinical trials showing evidence of a decrease in local recurrence but no survival benefit [44,45]. Several prospective and retrospective studies on selected patients with retroperitoneal sarcoma show that there may be a role for RT in local recurrence prevention [15,46,47,48]. The randomized controlled STRASS trial investigated the role of preoperative radiotherapy plus surgery versus surgery alone for primary retroperitoneal sarcoma, with the endpoint of abdominal recurrence-free survival (ARFS) [49]. The overall outcomes showed no difference in ARFS and no overall survival benefits at 3 years follow-up. On subgroup analysis by histological subtype, patients with well-differentiated liposarcoma showed an improvement in ARFS if they received preoperative RT. However, these results were limited by power and further investigation is needed to determine if retroperitoneal liposarcoma patients truly benefit from radiation therapy. The trial did find that radiotherapy plus surgery was associated with more adverse patient events. Ultimately, the results from the STRASS trial suggest that preoperative radiation therapy should not be a standard of care for primary RPS as there was no evidence of recurrence or survival benefit yet a high morbidity rate [49]. There has been recent investigation into the application of stereotactic body radiotherapy (SBRT) in the treatment of metastatic disease. SBRT utilizes high-precision treatment techniques to deliver a higher dose per fraction over a shorter treatment duration than standard fractionated courses. However, these studies have been limited to retrospective data with low power, with no randomized clinical control trials to date [50,51].

When deciding on using radiation, genetic conditions that predispose patients towards cancer development should be considered. There is a higher risk for radiation-induced malignancies, including sarcomas, in patients with retinoblastoma (RB1 mutation) or Li Fraumeni Syndrome (TP53 mutation) [52,53,54].

Radiation in the setting of multifocal metastatic disease is used for the palliation of symptoms such as pain, bleeding, or neurological complications, or to obtain local control on a progressing tumor. In the setting of oligometastatic recurrence, one can consider preoperative or postoperative radiation therapy if there is concern for positive margins, as there is the potential of slowing disease progression and development of recurrence at the site of metastasis. When surgery is not an option due unresectability (such as in instances where location is in relation to surrounding critical structures or due to limitations by the amount of bowel previously resected), co-morbidities or poor prognosis, radiation can be considered in the treatment of oligometastatic disease. However, the radiation oncologist and surgeon should work closely together, as the dose given in palliative treatments without the intent for further resection is typically different. These patients are best cared for at multidisciplinary sarcoma centers where tumor board discussions lead to treatment options available for the individual patient.

### 4.2. Chemotherapy

Sarcoma response to chemotherapy is historically poor which limits its role in the setting of primary disease. In the setting of advanced, recurrent and metastatic retroperitoneal sarcoma, anthracycline-based chemotherapy agents such as doxorubicin +/− ifosfamide are the most commonly used based on data from general soft tissue sarcoma trials and extremity sarcoma trials. While these therapies only provide about 20% disease control with no survival benefit in the setting of metastatic disease, chemotherapy is typically offered if the patient can tolerate the side effects [55]. In the setting of resectable oligometastatic disease, if the histology is high grade, a time course of chemotherapy may give insight into the disease progression before the risks of surgical resection are undertaken. While there have been limited clinical trials compared to other cancers due to the rarity of sarcoma, there have been new clinical trials in the past 5 years focused on select histologies and molecular subsets [56]. Some of these newer agents, such as trabectedin and eribulin, show only a modest progression-free survival benefit (4.2 months and 2.2 months, respectively) in patients who fail first and second line therapy [57,58]. For peritoneal metastasis, hyperthermic intraperitoneal chemotherapy (HIPEC) has been investigated with no evidence of benefit [59,60]. All patients with metastatic retroperitoneal sarcoma should be presented at multidisciplinary sarcoma tumor boards and offered a clinical trial, if eligible. This is the only way that we will continue to make progress and identify more effective systemic therapies for these patients.

### 4.3. Radiofrequency Ablation

Radiofrequency ablation (RFA) is an alternative therapy for the local control of isolated metastasis. There is a potential role for radiofrequency ablation in the treatment of isolated liver metastasis with palliative intent. However, ablative techniques and intra-arterial embolization (chemo-, bland-, and radio-embolization) have yet to show a significant impact on overall survival or improved outcomes in these patients [61]. The data are limited to small retrospective studies with less than 100 total patients, the majority of which with GIST tumors [62,63]. Discussions with interventional radiologists are encouraged for these patients, since ablative techniques allow an option for local therapy that is less morbid than surgical resection and can be performed with minimally invasive techniques. These procedures have been described as being used in a “test-of-time” approach, in which ablation can be performed for a resectable metastasis with a plan for metastectomy [64]. If patients are free of disease after ablation, they successfully avoided resection. On the other hand, if they develop extensive metastases after ablation, they would also avoid the morbidity of surgery which would have had no benefit [64]. There has yet to be a randomized control trial available analyzing the efficacy of ablative techniques in the treatment of oligometastatic retroperitoneal sarcoma. In centers that have expertise in RFA and other ablative techniques, these alternative therapies may be considered and discussed at a multidisciplinary sarcoma tumor board with interventional radiologists.

## 5. The “Carefully Selected Patient”

A common topic of discussion in sarcoma management is the qualifier of specific treatment options being available to the “carefully selected patient”. However, standardized selection criteria to quantify which patients this may apply to have yet to be created. This is because the “carefully selected patient” is dependent on complex, multifactorial criteria pertaining to each individual patient’s disease presentation and independent health and candidacy for the treatment options available (Table 2).

In terms of disease presentation in the evaluation of surgical intervention in the recurrent or metastatic setting, the carefully selected patient is one who has had a prolonged recurrence-free survival from the time of primary tumor resection to recurrence. Another factor is the presence of isolated or localized oligometastatic disease which would have a more meaningful treatment benefit than in the setting of multi-metastatic disease. In order to assess histology-specific retroperitoneal sarcoma recurrence and survival outcomes, the online validated nomogram “Sarculator” can be used to predict risk of recurrence and survival after the resection of recurrent RPS [10,13]. Patients with more aggressive histopathologies and/or widespread recurrence, and poor responses to systemic treatment, are less optimal candidates for resection as the intervention has a high morbidity with little impact on survival. If the patient has metastasis in more than one location, surgery can be considered for symptomatic palliative control, but will not likely improve life expectancy and must be considered with the impact on quality of life. In the carefully selected patient, surgery may lead to an improvement in overall survival (OS). In a retrospective study from the French Sarcoma Group, metastatic sarcoma patients who underwent locoregional therapies, including surgery, had an improved survival compared to those who had no locoregional therapies [65].

For the evaluation of patient candidacy for surgery in general, common perioperative evaluation includes baseline functional status, comorbidities, and nutritional status. Multiple studies have demonstrated a correlation between adequate perioperative nutrition and decreased complications [66,67]. Multiple indices exist that can be utilized in patient selection, including frailty and fragility indices, NISQIP, ASA, and Cardiac Risk calculators [68,69,70,71,72]. These tools predict the morbidity and mortality of the intervention but must be considered against the potential benefits of the surgery. In terms of metastatic retroperitoneal sarcoma, these operations can be very high risk, and the benefit is likely not in the form of cure but rather an increased life expectancy or a disease-free interval. These re-operations are complicated by adhesions, the increased risk of damaging surrounding structures, and the risk of short-gut syndrome if the small bowel was previously resected, additional to the specific risk of the targeted operation. The expected outcomes need to align with the individual patient’s goals.

## 6. Role of Palliative Surgery

In the carefully selected patient with oligometastatic disease, surgery is considered a treatment when the goal is to increase recurrence-free or progression-free survival. However, surgery may be offered for palliation when it is for symptom control without the specific intention of disease removal [73]. Palliative surgery should be considered for retroperitoneal sarcoma patients who experience symptoms such as severe pain or bowel obstruction, where surgical interventions such as diverting loop ileostomies or gastrostomy tubes (G-tubes) may improve quality of life without specifically treating the sarcoma [74]. The role of palliative surgery in metastatic disease is not well established and consideration needs to be given to potential complications of surgery in a patient in which the operation will not improve overall survival [75]. As such, debulking surgery has been shown to cause significant morbidity and mortality with little benefit and, therefore, is not recommended.

Metastatic retroperitoneal sarcoma is a disease with poor treatment options and a low likelihood of cure. There is a need for continuous shared decision making among patients and providers to ensure each patient’s goals of care are understood. The risks and side effects of treatments, both surgical and systemic, should be considered against the impact of taking no action on a patient’s mental health and mindset. In certain cases, the intervention may have a high risk of recurrence and failure, but this risk needs to be evaluated against the benefit of decreased mental burden on patients who feel a loss of hope when nothing is done. With retroperitoneal sarcoma, a disease with poor systemic options, it is important to have high-level discussions at multidisciplinary sarcoma tumor boards, where the options of systemic therapy versus local therapies, surgery and radiation are discussed to that the options presented to patients are reasonable and safe. When these high-failure treatments are considered, thorough discussions must be had with the patients and their families being very honest about the outcomes and expectations. The overall goal should be the best quality of life, and many times this means managing symptoms of metastatic disease while not using surgical interventions.

## 7. Conclusions

Surgery can be considered in the treatment of oligometastatic RPS in the “carefully selected patient” who has been presented and discussed at a multidisciplinary cancer center with sarcoma expertise [76,77]. This patient typically has limited disease, acceptable surgical risk, less aggressive histology, and had a longer time between primary resection and metastasis presentation. Given the limited number of randomized control trials regarding the treatment of RPS, selection bias is involved to best care for each patient, making it difficult to delineate true improvements with interventions versus carefully selected study populations. In order to improve the quality of care and quality of life of our RP sarcoma patients, there is a need for more clinical trials and well-planned prospective registry trials among sarcoma centers worldwide. With limited funding for these rare cancers, there continues to be a challenge to provide newer and improved treatment options. Multi-institutional and multi-national groups such as the Connective Tissue Oncology Society (CTOS, Alexandria, VA, USA) and The Transatlantic Retroperitoneal Sarcoma Working Group (TARPSWG) are bringing sarcoma researchers together to overcome these barriers.

## Figures and Tables

**Table 1:** Recurrence and survival outcomes of retroperitoneal sarcoma subtypes following primary resection.

Histologic Subtype	5-Year Local Recurrence Risk (%)	5-Year Distant Metastasis Risk (%)	10-Year Distant Metastasis Risk (%)	Most Common Location for Metastasis	Overall Survival ^a^
WDLPS	High (20–40%) [11,14]	Very Low (1–3%) [9,16]	Low (10%) [9,16]	Intra-Abdominal [17]	5-yr: 89% [18] 10-yr: 70% [11]
DDLPS	High (40–58%) [11,14]	Low for Grade I (5–10%) [16,19] Medium for Grade II–III (20–30%) [14]	Medium (30%) [11]	Intra-abdominal, Lungs [17]	5-yr: 44–50% [18,20] 10-yr: 30–50% [11,14]
LMS	Low (10–21%) [14,21]	High (47%) [14,21]	Very High (58%) [14]	Lungs [22]	5-yr: 60% [14,18] 10-yr: 40% [11]
UPS	High (40%) [14]	High (31–35%) [14,23]	High (50%) [24]	Lungs [23]	5-yr: 40–53% [14,23] 10-yr: 45% [24]
MPNST	Medium (20–35%) [11,14]	Low (15%) [14]	Low (15%) [14]	Lung [25]	5-yr: 51–65% [14,26] 10-yr: 45% [11]
SFT	Low (8–10%) [11,14]	Low (10%) [14]	High (40%)	Lungs, Liver [27]	5-yr: 80–84% [14,28] 10-yr: 70% [11]

^a^ From time of primary diagnosis.

**Table 2:** Key points to consider for surgical resection of oligometastatic disease.

Tumor Biology	Histology Grade Time to recurrence Location of metastasis
Patient ability to tolerate an operation	Fitness Frailty Comorbidities ASA status ^a^
Overall prognosis	Predicted overall survival Primary resection margin status Response to chemotherapy
Multidisciplinary tumor board discussion (radiation oncology, medical oncology, surgical oncology, radiology, pathology, and palliative care)	Risks versus benefits of all treatment options Clinical trial eligibility
Patient goals of care	Symptom management Quality of life

^a^ American Society of Anesthesiologists (ASA) physical status.
